# Device-Measured Physical Activity, Sedentary Behaviors, Built Environment, and Adiposity Gain in Older Women: A Seven-Year Prospective Study

**DOI:** 10.3390/ijerph18063074

**Published:** 2021-03-17

**Authors:** Pablo Molina-Garcia, María Medrano, Jana Pelclová, Izabela Zając-Gawlak, Lenka Tlučáková, Miroslava Přidalová

**Affiliations:** 1 Faculty of Physical Culture, Palacký University Olomouc, 779 00 Olomouc, Czech Republic; pablomolinag5@gmail.com (P.M.-G.); maria.medrano.echeverria@gmail.com (M.M.); miroslava.pridalova@upol.cz (M.P.); 2 The Jerzy Kukuczka Academy of Physical Education, 40-065 Katowice, Poland; i.zajac-gawlak@awf.katowice.pl; 3 Faculty of Sports, University of Presov, 080 01 Presov, Slovakia; lenka.tlucakova@unipo.sk

**Keywords:** accelerometer, fat mass, body mass index, neighborhood environment walkability scale

## Abstract

The search for determinants of adiposity gain in older women has become vitally important. This study aimed to (1) analyze the adiposity gain based on the participants’ age and (2) determine the prospective associations of baseline intrapersonal, built environment, physical activity, and sedentary behavior variables with the adiposity gain in older women. This was a seven-year prospective study (baseline: 2009 to 2012; follow-up: 2016 to 2019) in older women (*n* = 178, baseline age = 62.8 ± 4.1 years). Baseline and follow-up adiposity (bioelectrical impedance) and baseline physical activity, sedentary behavior (accelerometers), and intrapersonal and built environment (Neighborhood Environment Walkability Scale questionnaire) variables were included. The body mass index (BMI) increment tended to be inversely associated with the women’s age (*p* = 0.062). At follow-up, 48, 57, and 54% of the women had a relevant increase (d-Cohen > 0.2) in their BMI, percentage of body fat, and fat mass index, respectively. The women that spent ≥8 h/day being sedentary were 2.2 times (1.159 to 4.327 CI95%, *p* < 0.02) more likely to increase BMI (0.82 to 0.85 kg/m^2^) than non-sedentary women. No built environment variables were associated with seven-year adiposity gain (all *ps* > 0.05). A reduction in sedentary time should be promoted for adiposity gain prevention and health preservation in older women.

## 1. Introduction

The ageing of the world’s population will have an important influence on healthcare in the coming decades [1,2]. The prevalence of older adults who are overweight/obese has simultaneously considerably increased around the world [3]. Especially after menopause, women tend to experience a peak in their fat mass gain [4]. Excess adiposity increases the risk of developing cardiovascular diseases, type 2 diabetes, cancer, or mental diseases, among other morbidities and conditions in this population [5]. For this reason, we must detect the determinants, both interpersonal and contextual, of adiposity gain over time in older adults to enable its prevention or decrease.

Some longitudinal studies have analyzed the associations of intrapersonal (education and socio-economic information), built environment, and/or physical activity (PA) or sedentary behavior variables with adiposity in older adults [6,7,8,9,10,11,12]. Year after year, studies of the role of contextual factors in the progression of obesity have acquired increased importance in the scientific literature [12]. The built environment is defined as the human-formed, developed, and structured areas such as roads, utilities, or fixtures that form the physical characteristics of a place [13]. Although evidence exists on the longitudinal association between built environment variables and adiposity gain, the vast majority is focused on weight and body mass index (BMI) gain but not on other adiposity indicators such as fat mass percentage or fat mass index (FMI) [12]. The majority of the research examined the association of other environmental variables (e.g., food-related environment, and greenness) and were developed in middle-age adults [12]. Therefore, evidence of the longitudinal association of perceived environmental factors and adiposity gain in older adult populations is scarce [11,12].

Sedentary behavior has increased in the previous years due to changes in technology and the environment [10]. Sedentarism is nowadays considered the fourth cause of mortality around the world, with 3.2 million deaths per year [14]. Although sedentary behavior is related to a higher risk of metabolic morbidity and mortality, its association with weight gain during adulthood is unclear [10,15], especially among older adults. The literature reports different results regarding the longitudinal association between adiposity and PA. Whereas some of the studies found an inverse association between baseline PA and adiposity gain [16], others found that PA raised BMI [8], and others did not find this association at all [6,7]. When this association has been analyzed regardless sex, or in samples of women, the results differ between studies [8,16,17]. Therefore, this relationship in this population should be elucidated. Moreover, most of these longitudinal studies used self-reported PA or sedentary behavior [6,7,8], which are prone to both inaccurate reporting and introducing bias [18]. Conversely, accelerometers are demonstrated to be valid instruments for PA assessment in older adults [19], and the associations between physical activity and adiposity seem stronger when physical activity is measured by accelerometer compared with questionnaire self-reports [20].

Therefore, the aims of the present study were (1) to analyze the increase in adiposity based on the age of participant women, and (2) to determine a prospective association of intrapersonal, built environmental and device-measured PA and sedentary behavior variables at baseline with relevant adiposity gain during a seven-year follow-up period in older women. Considering previous findings related to movement behaviors in older adults, we hypothesized that older women being more active and less sedentary at baseline would be less likely to have a relevant gain in adiposity after the seven-year follow-up period.

## 2. Materials and Methods

### 2.1. Study Design and Population

This study was a prospective investigation in older women in three central European cities with very similar economic, cultural, and weather conditions. These cities were Olomouc in the Czech Republic, Katowice in Poland, and Prešov in Slovakia. A detailed description of the study was published elsewhere [21]. Participants for the baseline stage (2009 to 2012) were women with mean age 62.8 ± 4.1 years attending the University of the Third Age. Inclusion criteria for enrolment to the study were being aged 60 years onward, living independently, and being able to walk without any prosthetic aids. Follow-up stage assessments were scheduled individually with each participant between 2016 and 2019 to ensure a seven-year period. Participants were assessed in all measurements both at baseline and at seven-year follow up. For baseline and follow-up assessments, the participants were informed that their participation was voluntary and that they could withdraw from the study at any time. For both stages, they provided written consent. The prospective study was approved by the Institutional Research Ethics Committee, Faculty of Physical Culture, Palacký University Olomouc under reference No. 20/2017 and was carried out in accordance with the Declaration of Helsinki.

Of 409 eligible older women at baseline, 42 died, 72 refused to participate again, 65 were ineligible, and 28 had no valid contact. Thus, the follow-up sample consisted of 202 women. Of these, 19 women did not have valid data from the accelerometer and five women did not have valid data from body composition assessment. Hence, 178 women were included in the final analyses.

### 2.2. Adiposity Indicators

Percentage of fat mass (FM%), fat mass index (FMI), and BMI were used as indicators of adiposity. Fat mass was assessed by multi-frequency bioelectrical impedance analysis using the InBody 720 device (Biospace Co., Ltd., Seoul, Korea) under laboratory conditions according to the tool manual instruction and as was reported elsewhere [22]. Both at baseline and follow-up periods, all adiposity indicators were measured one time in the morning, wearing light-weight clothes and barefoot [22]. The multi-frequency bioelectrical impedance analysis has been suggested as a valid method for the assessment of adiposity in our target population, regardless of body weight and physical activity level [23]. The same device was used to measure body weight to the nearest 0.1 kg. In advance, the participating women were asked to avoid any vigorous activity for at least 48 h and to fast for at least 2 h. Body height was measured using the P-375 portable stadiometer to the nearest 0.1 cm (Trystom, Olomouc, Czech Republic). BMI and FMI were calculated as weight/height^2^ and fat mass/height^2^, respectively.

Then, women were categorized as maintain/decrease or increase in BMI, FM%, or FMI according to an individual change of <0.2 or ≥0.2 of the Cohen’s d, respectively. A change of 0.2 was selected as the minimum value for a relevant adiposity increase during the seven-year follow-up period [24]. A d-Cohen effect size ≥0.2 was usually accepted as a minimum change and was previously employed by other authors to define the minimum relevant changes in prospective analyses [25].

### 2.3. Intrapersonal and Built Environmental Variables

Intrapersonal variables were self-reported and obtained at baseline and follow-up. Satisfaction with life was obtained from the Satisfaction with Life Scale [26], dichotomized as high satisfaction or low satisfaction (neutral or dissatisfied). Education level was dichotomized as primary/secondary education or tertiary degree. Employment status was categorized as employed, unemployed, or retired. Marital status was dichotomized as living alone or living with a partner.

A modified and validated culturally adapted abbreviated version of the Neighborhood Environment Walkability Scale (NEWS-A) questionnaire was used to obtain data about the perceived environment including categories such as residential density, land use mix (including both indices of proximity and accessibility), street connectivity, infrastructure for walking/cycling, neighborhood aesthetics, and traffic and crime safety [27]. For each environmental category, the final score was transformed into a categorical variable with two levels: low and high perception of the environment. The cut-off points for these levels were set according to the median of the sample (land use mix proximity: low < 3, high ≥ 3; land use mix accessibility: low ≤ 3, high > 3; street connectivity: low ≤ 3, high > 3; infrastructure for walking/cycling: low ≤ 2.5, high > 2.5; neighborhood aesthetics: low ≤ 2.5, high > 2.5; traffic and crime safety: low ≥ 2, high < 2; residential density: low < 167, high ≥ 167).

### 2.4. Physical Activity and Sedentary Behavior

For baseline and follow-up, an ActiGraph GT1M accelerometer (Manufacturing Technology Inc., Pensacola, FL, USA) was used for PA and sedentary behavior assessment. The participants were asked to wear the device on their right hip during waking hours for at least seven consecutive days. The sampling time interval was set to 60 s and non-wear time was defined using the Troiano algorithm [28]. The inclusion criteria for the final analyses were ≥10 h of daily wear time and ≥4 days (three workdays and one weekend day). Counts and step counts were derived for each day from the manufacture’s software (ActiGraph, LLC, Pensacola, FL, USA). The low frequency filter was not used. For sedentary behavior, light intensity PA (LPA), and moderate-to-vigorous PA (MVPA), the cut offs were defined as ˂100 [29], 100 to 1951 [30], and ≥1952 counts/min [30], respectively. Women having ≥8 h/day of sedentary behavior were classified as sedentary [31]. According to the MVPA, women were categorized as not meeting 150 min/day, meeting 150 to 300 min/day, and exceeding 300 min/day [32]. Moreover, ≥10,000 steps/day was the cut-off for meeting step-based recommendations [33]. Participation in any organized PA in a week was self-reported and obtained at baseline.

### 2.5. Statistical Analysis

The data are presented as means (standard deviations) or absolute and relative prevalence (N, %), unless otherwise indicated. Differences between the baseline and follow-up adiposity indicators were analyzed by the dependent *t* test.

Before performing analyses, data were examined to detect extreme values and were winsorized (i.e., ΔBMI = 1, ΔFM% = 1, ΔFMI = 1) to limit their influence on the analyses. Likewise, dummies from categorical variables were calculated to include them in the analyses (i.e., MVPA categories). All continuous variables followed a normal distribution according to the verification of skewness and kurtosis values. The relationship between women’s age at baseline and adiposity gain was determined using linear regressions. Logistic regressions were conducted to determine the influence of baseline intrapersonal, built environment, and device-measured PA and sedentary behavior variables on the likelihood for a significant increase in BMI, FM%, or FMI during the seven-year follow-up period. Country (as a dummy variable), age at baseline, follow-up duration, and accelerometer wear-time (only for device-measured PA and sedentary time variables) were entered as confounders into all the analyses. All statistical analyses were performed using version 23.0 of the Statistical Package for the Social Sciences (SPPS Inc., Chicago, IL, USA). The level of significance was set at α = 0.05.

## 3. Results

The adiposity, intrapersonal, perceived environmental, and PA and sedentary behavior baseline characteristics, as well as the adiposity changes of all woman participating in the study, are described in Table 1. At baseline, 178 women (62.8 ± 4.1 years) participated in the study, of which 48% had primary education, 71% lived with a partner or were married, and 73% were retired. Almost half of the woman were sedentary and spent ≥8 h/day sedentary, whereas more than two-thirds reached ≥150 min/day of MVPA or participated in organized PA, and more than half accumulated ≥10,000 steps/day. In these women, the mean of the adiposity gain after seven years was 0.9 ± 1.7 kg/m^2^, 2.4 ± 3.7%, and 1.0 ± 1.6 kg/m^2^ for BMI, FM%, or FMI, respectively.

**Figure 1:**
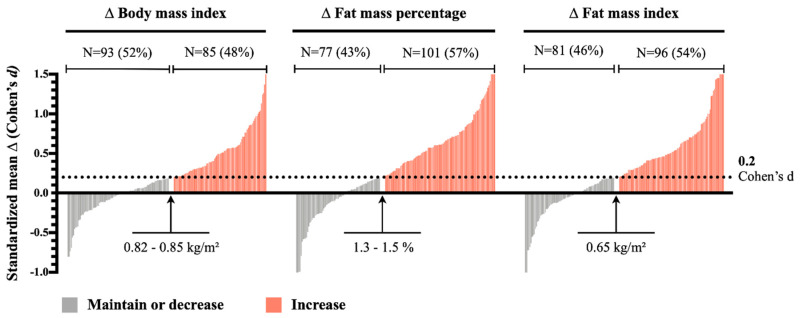
Distribution of participants according to the change of body mass index, fat mass percentage, and fat mass index and their classification according to the maintenance or decease (Cohen’s d < 0.2) or a minimum significant increase (Cohen’s d ≥ 0.2) in the adiposity variables after the seven-year follow-up. Values are absolute and relative prevalence (%).

Figure 1 depicts the interindividual variability in BMI, FM%, and FMI changes during the seven-year follow-up period. During this follow-up period, 85 (48%), 101 (57%), and 96 (54%) of women had a relevant increase in BMI, FM%, or FMI, respectively.

**Figure 2:**
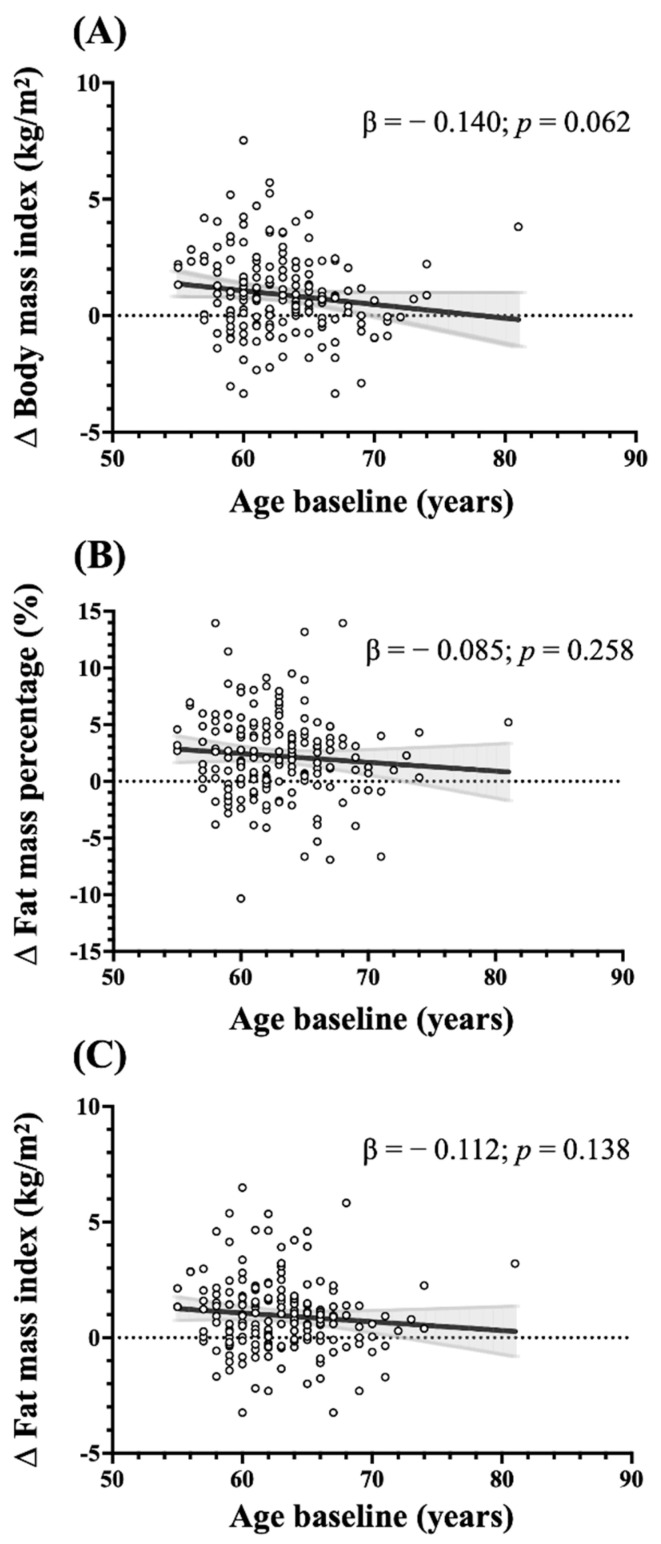
Distribution of participants according to the change of (**A**) body mass index, (**B**) fat mass percentage and (**C**) fat mass index after the seven-year follow-up. Values are absolute and relative prevalence (%).

Figure 2 shows the changes in adiposity in relation to the age of the participants at baseline. The mean change of adiposity was higher than zero (which indicates an increase) in all years. Although it was not significant (*p* < 0.07), the increase in BMI was inversely associated with the age of the women; BMI in younger women increased more than in older women during the seven-year follow-up period.

**Figure 3:**
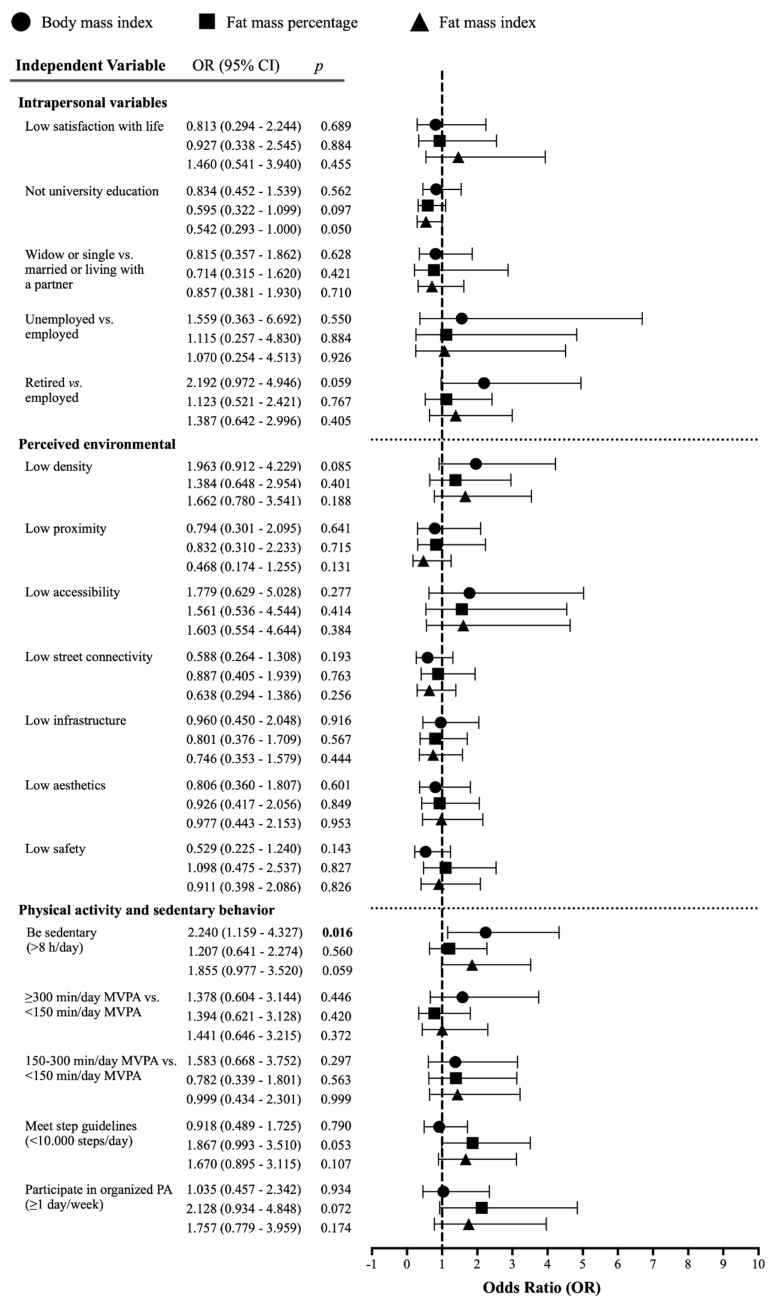
Forest plot showing the likelihood of a minimum relevant increase in body mass index (circles), fat mass percentage (squares), and fat mass index (triangles) during the seven-year follow-up according to intrapersonal, environmental, and device-measured PA and sedentary behavior variables at baseline. Boldfaced values: *p* < 0.05. Univariate logistic regressions were adjusted by country (dummy), baseline age, and follow-up duration. PA and sedentary behavior variables were additionally adjusted for accelerometer wear time. Abbreviations: OR: odds ratio; PA: physical activity; MVPA: moderate-to-vigorous physical activity.

The prospective association of intrapersonal, built environmental, or objectively measured PA and sedentary behavior variables and the likelihood of having a relevant gain in adiposity during the seven-year follow-up period is shown in Figure 3. We observed that sedentary women had a >2.2 times higher likelihood (*p* < 0.02, Figure 3) of having a relevant BMI gain (0.82 to 0.85 kg/m^2^) in the seven years than non-sedentary women. No further intrapersonal, built environmental, or device-measured PA and sedentary behavior variables increased the likelihood of a relevant adiposity gain in the seven-year follow-up period.

## 4. Discussion

The main findings of the present study are that during this follow-up period, approximately half of women had a relevant increase in BMI, FM%, or FMI, and that sedentary women (those that had more than eight hours per day of sedentary behavior) were more likely to experience a relevant increase in BMI after a seven-year follow-up period. Due to the increase in adiposity gains and the associated health problems, these results emphasize the importance of the development and implementation of strategies aimed to reduce sedentary behavior to preserve the health of older women. Intrapersonal variables, built environment, or PA did not demonstrate any prospective associations with adiposity changes during the seven-year follow-up period.

Women participating in this study gained BMI, FM%, and FMI at seven-year follow-up, although it attenuated with increasing age. Fat mass and weight gain demonstrated a trend of increasing through the lifespan until a certain time, somewhere between 60 and 80 years, at which it stabilizes or decreases [34]. In our study, participants were not yet in a decrease phase, so weight and fat mass gain prevention strategies would have been needed.

Our findings do not prove that intrapersonal or perceived environmental factors influenced relevant changes in the adiposity of our sample. The available literature on this topic evidence a lack of strong associations of environmental factors with obesity indicators, mainly due to methodological limitations such as the lack of longitudinal studies allowing the inference of causality [35]. A recently published review of longitudinal studies identified 33 articles examining the neighborhood effects on obesity outcomes in the adult population [12]. Although the large variety of indicators hampered comparison between studies and drawing conclusions, 65% of included articles reported non-significant associations or mixed effects, while only 35% found significant associations. This discrepancy could be partially explained by methodological differences such as sample characteristics, the instruments used to measure adiposity or intrapersonal/environmental factors, or the statistical analyses. Therefore, more research with similar approaches should be performed to elucidate these findings. Specifically, in our study, the fact that our sample is composed only of women with similar socioeconomic status, high accessibility, street connectivity, and good walking/cycling infrastructure could explain the lack of influence in adiposity changes. Importantly, only 4.8% of the longitudinal analyses in this review included perceived environmental variables in the study of obesity indicators, which was one of the aims in the present study [12]. Other novelties of our results are the inclusion of a homogeneous sample of elderly women from three central European countries, knowing that the vast majority of studies focused on young or middle-aged adults, and the study of additional adiposity indicators, since 98% of previous articles only included BMI, weight, or waist circumference [12].

Both sedentary behavior and PA are considered essential to understanding changes in adiposity over time, especially due to their role in the energy balance [36]. It is therefore unsurprising to find a huge body of evidence on this topic, although strong debate remains ongoing about the real effects of sedentary behavior and PA in weight and adiposity gain. One of the main limitations of previous studies is that the vast majority used self-reported measures of PA and sedentary behavior, which are generally highly biased [37]. The United States Physical Activity Guidelines Advisory Committee (PAGAC) recently reviewed the literature to guide PA intervention in the promotion of weight loss/maintenance and found only three studies (of a total of 33) using measures of PA via accelerometers or pedometers [38]. For instance, a validation study comparing PA questionnaires against accelerometers observed a mean underestimation of 12 min per day of MVPA [39], a difference that impacts the association with body composition, as demonstrated by recent studies [20]. All this reinforces the need for further studies investigating the longitudinal association of device-measured PA with adiposity changes, as we did in our population of older women.

Recently, an update from the 2018 PAGAC reported limited evidence on the longitudinal association between sedentary behavior and weight status [40]. The available literature is contradictory, with some authors reporting that sedentary behavior is associated with increases in adiposity over time [41], others finding no associations with obesity degree or weight gain [42,43], and others indicating unclear findings [44]. All these studies used self-reported sedentary behavior, whereas the only available evidence using device-based measures, and therefore the most comparable to the present study, found no associations of sedentary behavior with subsequent weight gain in Norwegian middle-aged adults [45]. Notably, all these studies included a more heterogeneous and younger sample than ours. Therefore, our results help provide evidence on this topic and suggest that, in a particular sample of older women from central Europe, those with at least eight hours of sitting time per day are more likely to experience an increase in their BMI. More research that includes all the adiposity variables, and not only weight or BMI, should be performed to elucidate differences in the associations of BMI and the other adiposity variables.

Our results showed no influence of PA on BMI changes after the follow-up period. With regard to BMI, a recent systematic review included 40 articles that longitudinally studied the role of overall PA in the prevention of BMI and weight gain in adults of different ages [46]. These authors found strong evidence of the relationship between PA and attenuated weight gain, establishing the critical exposure as above 150 min per week, with special emphasis on MVPA [46]. However, this association demonstrated an overall attenuation with increasing age, which indicated that PA may be more effective in weight gain prevention for young and middle-aged adults than for older adults. The same findings have been observed in two relevant cohort studies, demonstrating that PA attenuated weight gain in women until 60 to 65 years, a turning point at which PA appears to be less effective for this purpose [17,47]. Nevertheless, contradictory results exist in the literature, since not all studies found this attenuation in older adults [16,48,49]. In the only longitudinal study including device-measured PA in a population of elderly adults, baseline PA did not predict weight gain in a six-year follow-up period. Overall, the present study adds to the available literature by suggesting that PA does not influence BMI gain in older women.

The longitudinal association of PA with other adiposity gain variables has been considerably less studied in the literature in comparison with body weight, mainly due to being a less affordable measure. In the present study, we found that the PA level at baseline was not related to relevant changes in FM% or FMI. Although higher levels of PA generally prevent fat mass accumulation, most of the available studies focused on young and middle-aged adults; research on older adults is scarce [41]. Sims et al. [17] found that higher levels of PA attenuated fat mass gain over a six-year follow-up in women aged 50 to 69 years old, whereas women 70 years old onward demonstrated a fat mass loss independent of their PA level. Similarly, Ragusso et al. [7] reported that leisure-time PA did not prevent fat mass accumulation in adults over 65 years old during a follow-up of three years. Laddu et al. [50] found a relevant role of PA by demonstrating in men over 65 years old that those performing less moderate-intensity activities experienced a significant increase in fat mass after a five to seven-year period of follow-up. As can be observed, the literature is contradictory in older adults and further longitudinal studies should be conducted to elucidate whether PA plays a significant role in the prevention of adiposity gain at this specific stage of adulthood.

There are several strengths that should be highlighted in the present study. One of the main strengths is the inclusion of device-based measures of PA and sedentary behavior through ActiGraph GT1M accelerometer devices, thus overcoming one of the most common limitations observed in this research [38]. Apart from BMI, we reported additional adiposity indicators such as fat mass percentage and FMI, which have been less studied in longitudinal research with environmental and PA factors [12,38]. Changes in adiposity were classified according to relevant changes (i.e., Cohen’s d), which avoids a substantial loss of information in comparison with traditional overweight/obese classifications and provides information about the interindividual variation in our sample [38]. We provided a multidimensional perspective on this research topic by integrating a wide variety of intrapersonal, built environmental, and PA and sedentary behavior variables together that have been related to adiposity gain [51]. Our homogeneous sample of older women allowed us to focus on a population that has received less attention in the literature and that is experiencing a worrying increase in adiposity [52].

However, our findings come with several limitations that should be considered in their interpretation. First, the observational design of the study, even longitudinal, prevents the establishment of causality. Second, our analyses only included baseline predictor variables (i.e., environmental and PA and sedentary behavior), which explain how having a specific behavior in a time point influences the adiposity gain in the follow-up period (independent of whether the behaviors change or not in the follow-up period), but not the association between the changes in both variables. Third, we did not include dual-energy X-ray absorptiometry (DXA) as the gold standard to measure adiposity, although bioelectrical impedance was found suitable for our target population [21]. Fourth, accelerometers are not totally accurate yet in identifying sedentary behavior; there was the possibility that slight misclassifications were present. Even so, accelerometers are more trustful than self-reported measures. Fifth, our findings may be confounded by unmeasured factors such as medication or comorbidities. Lastly, our sample was relatively small and cannot be considered representative of older women from central European countries. Furthermore, relevant changes in adiposity were calculated according to this sample, which limits the extrapolation of our results to other populations.

## 5. Conclusions

A relevant seven-year increase in adiposity was found in approximately half of older women. Device-measured sedentary behavior influenced BMI gain over a follow-up period of seven years in older women from central Europe. The women who were sedentary for at least eight hours per day were more likely to have a relevant BMI increase during a seven-year follow-up period. No prospective associations with adiposity changes during the seven-year follow-up period were found for intrapersonal, built environment, or PA variables.

The results of this study highlight the importance of performing health strategies to reduce sitting time in older women to prevent adiposity gain and thereby preserve their health.

## Figures and Tables

**Table 1:** Description of the baseline characteristics and adiposity changes of all the women participating in the prospective seven-year follow-up study.

	*n*	Baseline	Seven-Year Follow-Up	Change (Follow-Up Minus Baseline)
Age (years)	178	62.8 (4.1)		
**Adiposity indicators**				
BMI (kg/m^2^)	178	26.3 (4.1)	27.2 (4.3)	0.9 (1.7) *
Fat mass (%)	178	34.4 (6.7)	36.6 (6.8)	2.4 (3.7) *
FMI (kg/m^2^)	178	9.3 (3.1)	10.2 (3.3)	1.0 (1.6) *
**Intrapersonal**				
Low satisfaction with life (N, %)	114	20, 18		
Education				
Primary (N, %)	178	85, 48		
University studies (N, %)	178	93, 52		
Family status				
Married or living with a partner (N, %)	122	87, 71		
Widow or single (N, %)	122	35, 29		
Employment				
Retired (N, %)	176	129, 73		
Employed (N, %)	176	36, 21		
Unemployed (N, %)	176	11, 6		
**Perceived environmental**				
Low density	125	63, 50		
Low proximity (N, %)	82	31, 38		
Low accessibility (N, %)	74	22, 30		
Low street connectivity (N, %)	125	42, 34		
Low walking/cycling infrastructure	125	42, 34		
Low aesthetics (N, %)	125	52, 42		
Low safety (N, %)	125	64, 51		
**Physical activity and sedentary behavior**				
Sedentary behavior (min/day)	178	437 (85)		
LPA (min/day)	178	369 (83)		
MVPA (min/day)	178	41 (24)		
Total PA (counts/min)	178	400 (124)		
Step count (steps/day)	178	9817 (2976)		
Be sedentary (≥8 h ST/day)	178	86, 48		
Meet PA recommendations				
No: ≤150 min/day MVPA (N, %)	178	39, 22		
Yes: 150–300 min/day MVPA (N, %)	178	61, 34		
Yes: ≥300 min/day MVPA (N, %)	178	78, 44		
Meet step recommendations (N, %)	178	102, 57		
Participation in organized PA (N, %)	125	88, 70		

Continuous variables were represented as means (standard deviations) and categorical variables as N, % absolute, relative prevalence). * = Difference between baseline and follow-up *p* < 0.001 (by dependent t test). Abbreviations: LPA, light physical activity; MVPA, moderate-to-vigorous physical activity; BMI, body mass index; FMI, fat mass index. Missing data in satisfaction in life, family status, participation in organized PA and all perceived environmental variables were due to incomplete self-reported data at baseline.

## Data Availability

The data presented in this study are available in the Figshare repository, https://doi.org/10.6084/m9.figshare.14223014.v1.
